# Synthetic Collagen-like Polymer That Undergoes a Sol–Gel Transition Triggered by *O–N* Acyl Migration at Physiological pH

**DOI:** 10.3390/ijms23031584

**Published:** 2022-01-29

**Authors:** Shinichiro F. Ichise, Takaki Koide

**Affiliations:** 1 Waseda Research Institute for Science and Engineering, Waseda University, Tokyo 169-8555, Japan; k2nt1@akane.waseda.jp; 2 Department of Chemistry and Biochemistry, School of Advanced Science and Engineering, Waseda University, Tokyo 169-8555, Japan

**Keywords:** extracellular matrix, collagen, peptide, three-dimensional cell culture

## Abstract

We previously reported an artificial collagen gel that can be used as a cell-culture substrate by end-to-end cross-linking of collagen-like triple-helical peptides via disulfide bonds. However, the gel had to be formed a priori by polymerizing the peptide in an acidic solution containing dimethyl sulfoxide for several days, which prevented its use as an injectable gel or three-dimensional (3D) scaffold for cell culture. In this study, we developed a collagen-like peptide polymer by incorporating an *O–N* acyl migration-triggered triple helix formation mechanism into a collagen-like peptide, which formed a gel within 10 min. We demonstrated that the collagen-like peptide polymer can be used as a 3D cell scaffold and that the 3D structure formation of cells can be controlled by collagen-derived bioactive sequences introduced into the peptide sequence.

## 1. Introduction

Collagen is the main structural protein in the extracellular matrix, and it forms supramolecules, such as fibrillar and reticular structures, in the body. The collagen molecule has a right-handed triple-helical structure composed of three polypeptide chains [1]. Each polypeptide chain in the triple-helical domain of collagen has Xaa-Yaa-Gly repeats. Specifically, fibrillar collagen has approximately 340 tandem repeats of Xaa-Yaa-Gly. The amino acids in Xaa and Yaa are often occupied by Pro and 4-hydroxyproline (Hyp/O) residues, respectively, and these imino acid residues increase the thermal stability of the triple helix [2,3,4]. Isolated fibrillar collagen self-assembles into fibrils and then fibers under physiological conditions in vitro, and it then forms a gel.

Collagen not only functions as a scaffold for cells, but it also functions as a bio-signaling molecule that regulates cell survival and differentiation by interacting with membrane receptors and soluble proteins. The amino acid sequences recognized by collagen-binding macromolecules have been identified. Collagen-binding integrins α1β1, α2β1, α10β1, and α11β1 mediate cell adhesion, survival, and proliferation. The Gly-Phe-Hyp-Gly-Glu-Arg (GFOGER) sequence on the collagen triple helix has been identified as a high-affinity site for integrin α2β1, a major collagen-binding integrin [5,6]. The collagen-binding receptor tyrosine kinases, discoidin domain receptor (DDR) 1 and 2, also regulate cell adhesion, survival, and proliferation. DDRs recognize Gly-Val-Met-Gly-Phe-Hyp on the collagen triple helix [7]. Both the von Willebrand factor (VWF) for blood coagulation and secreted protein acidic and rich in cysteine (SPARC) bind to Arg-Gly-Gln-Hyp-Gly-Val-Met-Gly-Phe containing the DDR-binding sequence [8,9,10]. The pigment epithelium-derived factor (PEDF) and heparan sulfate proteoglycans in syndecans competitively bind to Lys-Gly-His-Arg-Gly-Phe-Ser-Gly-Leu on the collagen triple helix and regulate angiogenesis [11,12,13].

Collagen is a useful biomaterial in research and medical applications because of its polymeric properties and biological activities [14]. Recently, the development of chemically synthesized artificial collagen as a collagen surrogate has been reported [15,16]. Artificial collagen is a promising biomaterial that can provide matrices with specific biological activities by incorporating bioactive sequences.

Peptides with tens of amino acid residues consisting of tandem Xaa-Yaa-Gly repeats (collagen-like peptides) form the collagen-like triple helix by self-assembly. Several artificial collagens that can form a gel by the supramolecular formation of collagen-like peptides have been reported. A trimeric collagen-like peptide in which three peptide strands are staggered and tethered by disulfide bonds forms a gel by elongating the triple helix [17,18,19]. Cell adhesion activity has been introduced into the artificial collagen-like material by incorporating the integrin-binding GFOGER sequence [20]. However, it is not suitable for practical use because the need for the stepwise reaction of disulfide bond formation results in high synthetic cost. Collagen-like peptides consisting of Pro-Hyp-Gly repeats with Lys and Asp residues in appropriately designed positions form a gel by elongation of the triple helix through sticky-end assembly and further lateral interactions via salt bridges [21,22]. However, rearrangement of the acidic and basic amino acids in the peptide sequence is critical for supramolecular formation. Therefore, the amino acid sequence can hardly be rearranged, and it is difficult to incorporate bioactive sequences into the peptide sequence.

Recently, we developed another artificial collagen system by end-to-end cross-linking of collagen-like triple-helical peptides [23]. We found that a peptide with two or more Cys residues at each terminus of the collagen-like sequence formed a gel by triple helix formation and subsequent cross-linking via disulfide bonds. The polymer gel exhibited integrin-dependent cell attachment activity by incorporating the responsible amino acid sequence into the peptide. The cell attachment activity was tuned by altering the concentration of the binding sequence in the polymer. The stiffness of the gel could also be tuned by altering the number of Cys residues. However, the preparation process requires more than 3 days of oxidation in a solution containing 10% (*v*/*v*) dimethyl sulfoxide (DMSO) as an oxidant [24], and physical destruction of the gel is irreversible. Therefore, the gel has to be formed into a shape a priori, and it is difficult to be applied to a 3D scaffold for cell culture or an injectable gel for medical use.

In this study, we developed a collagen-like peptide polymer that undergoes a rapid sol–gel transition in situ triggered by a simple pH shift. The transition mechanism is based on *O–N* acyl migration of an *O*-acyl isopeptide unit in the collagen-like peptide followed by triple helix formation. The *O*-acyl isopeptide moiety in the peptide is stable under acidic conditions, and it is converted to the corresponding amide form through *O–N* acyl migration at physiological pH. This mechanism allows the pH-triggered β-sheet formation of the amyloid peptide [25,26,27]. In a previous study, we found that a glycylserine *O*-acyl isopeptide unit incorporated into a collagen-like peptide sequence prevented triple helix formation under acidic conditions and induced triple helix formation by conversion to the amide form at pH 7.4 [28].

## 2. Results and Discussion

### 2.1. Design of a Collagen-like Peptide Polymer That Forms a Gel In Situ through a Sol–Gel Transition

**Figure 1:**
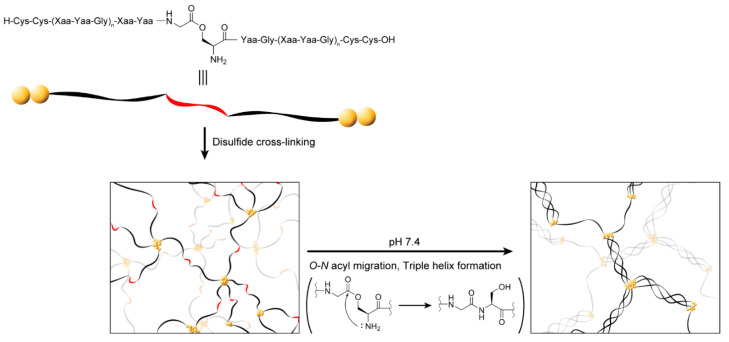
Concept of formation of a gel from a collagen-like peptide polymer by *O–N* acyl migration. The collagen-like peptide, which has a glycylserine *O*-acyl isopeptide at the center of the sequence and two Cys residues at each terminus, is polymerized by cross-linking via disulfide bonds and forms a gel by triple helix formation indued by *O–N* acyl migration at physiological pH.

We devised a strategy to form a gel from a collagen-like peptide polymer by triple helix formation induced by pH-triggered *O–N* acyl migration at physiological pH, as shown in Figure 1. This peptide was composed of collagen-like Xaa-Yaa-Gly repeats with two Cys residues at each terminus and a glycylserine *O*-acyl isopeptide in the center of the sequence. The peptides were cross-linked via disulfide bonds under acidic conditions. The sol–gel transition was expected to be induced by triple helix formation triggered by *O–N* acyl migration at physiological pH.

### 2.2. Synthesis and Structural Analysis of the Collagen-like Peptide Containing an O-Acyl Isopeptide Unit

We designed a peptide consisting of 12 repeats of Xaa-Yaa-Gly with 2 Cys residues at each terminus and a glycylserine *O*-acyl isopeptide unit at the middle of the sequence (**C2-ester**, Table 1). The peptides were constructed by 9-fluorenylmethoxycarbonyl (Fmoc)-based solid-phase synthesis using *N*,*N*′-diisopropylcarbodiimide (DIC), and 1-hydroxybenzotriazole (HOBt) as coupling agents. To incorporate the *O*-acyl isopeptide unit and Pro-Hyp-Gly repeats, Boc-Ser(Fmoc-Gly)-OH and Fmoc-Pro-Hyp-Gly-OH were used as building blocks, respectively. The peptides were cleaved from the resin and deprotected by a trifluoroacetic acid (TFA)-based cocktail, and they were purified by reverse-phase high-performance liquid chromatography (RP-HPLC). The results of RP-HPLC and mass spectrometry of the purified peptides are shown in Figures S1 and S2. The amide form peptide corresponding to the expected product of *O–N* acyl migration of C2-ester was also independently prepared by the same procedure (C2-amide, Table 1).

**Figure 2:**
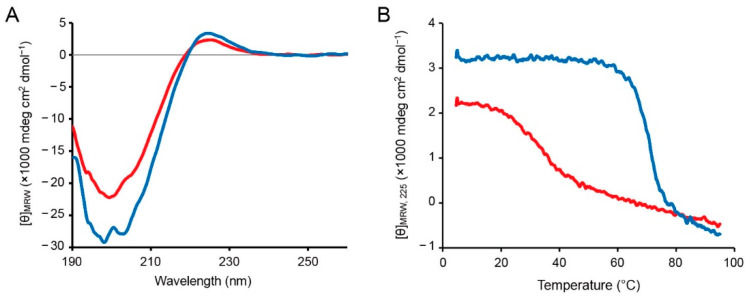
Conformational analysis of the peptides by CD spectroscopy. (**A**) CD spectra of C2-ester and C2-amide at 4 °C. The peptides were annealed in 0.05% (*v*/*v*) TFA(aq), and the spectra were measured in the presence of 10 mM TCEP. (**B**) Monitoring of the CD signal at 225 nm with increasing temperature from 4 to 95 °C. The red and blue lines are C2-ester and C2-amide, respectively.

Circular dichroism (CD) analysis was performed to confirm that the *O*-acyl isopeptide unit disturbed triple helix formation, but the amide form peptide can form the triple helix. C2-ester and C2-amide were dissolved in 0.05% (*v*/*v*) TFA(aq) and then annealed at 4 °C overnight after heating at 95 °C. The CD spectra at 4 °C were measured (Figure 2A). Both C2-ester and C2-amide showed a maximum signal at around 225 nm and a minimum signal at around 200 nm, indicating the polyproline-II-like secondary structure. The signals of C2-ester were smaller than those of C2-amide, indicating that C2-amide had higher helicity. In addition, the signal at 225 nm was monitored with increasing temperature (Figure 2B). The signal cooperatively decreased at around 30 °C for C2-ester and around 70 °C for C2-amide, and C2-ester showed a more gradual decreasing signal. The cooperative decrease in the signal at 225 nm is a typical property of the collagen-like triple helix, strongly suggesting that both C2-ester and C2-amide formed the triple helix [29].

The results also suggested that although C2-ester contained triple-helical population, the triple helix was disturbed and destabilized by the *O*-acyl isopeptide unit interrupting the continuous Xaa-Yaa-Gly sequence. In addition, the signal at 225 nm more gradually decreased with increasing temperature for C2-ester than for C2-amide, suggesting that the triple-helical population of C2-ester was more heterogeneous.

### 2.3. Investigation of Gel Formation of the C2-Ester Polymer Induced by O–N Acyl Migration

**Figure 3:**
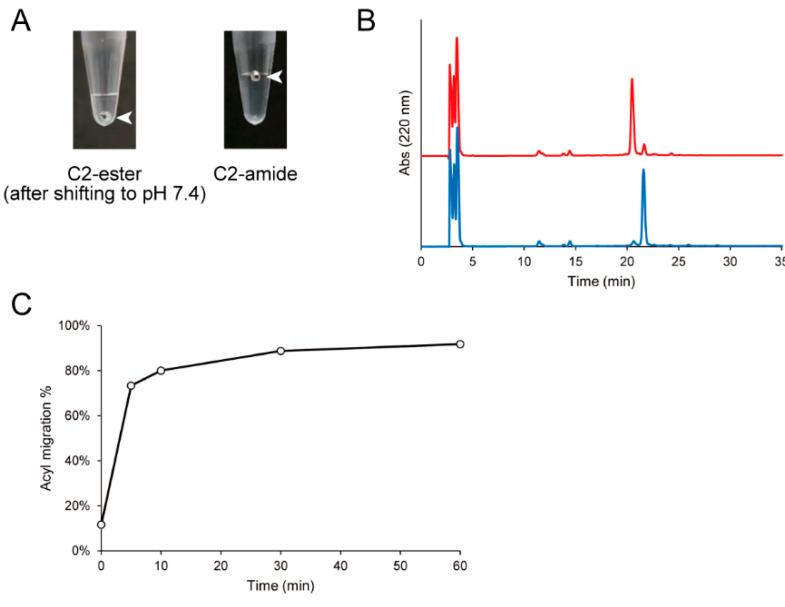
Evaluation of gel formation and *O–N* acyl migration of the C2-ester polymer. (**A**) Evaluation of gel formation of the C2-ester polymer after addition of 10× PBS. Gel formation of the C2-ester polymer was evaluated after the addition of 10× PBS to the polymer solution at the final concentration of 10 mg/mL followed by incubation at 37 °C for 1 h. A stainless-steel ball was set on the interface of the polymer, and gel formation was judged by whether the ball was held on the interface. The C2-amide polymer polymerized in a solution containing 10% (*v*/*v*) DMSO and 0.05% (*v*/*v*) TFA at a peptide concentration of 10 mg/mL was also evaluated as a control. The arrowheads indicate the positions of the stainless-steel balls. (**B**) RP-HPLC analysis of the reduced C2-ester polymer before (red) and after (blue) PBS treatment. The C2-ester polymer solution was subjected to the addition of 10× PBS at a final polymer concentration of 10 mg/mL and incubated at 37 °C for 1 h. The solution was reduced by TCEP in 0.05% (*v*/*v*) TFA(aq) and then analyzed by RP-HPLC with a linear gradient of acetonitrile/water containing 0.05% (*v*/*v*) TFA of 10%–20% in 30 min. The peaks at 21 and 22 min were the C2-ester and corresponding amide-form compound, respectively. (**C**) Quantitation of *O–N* acyl migration in the C2-ester polymer. The percentage of acyl migration was calculated by the ratio of the amide form in the total peak area of the ester form and the amide form in HPLC analysis.

First, we investigated whether C2-ester was in the sol state after oxidative polymerization and whether the polymer sol can be transformed to a gel by increasing the triple helicity by *O–N* acyl migration at physiological pH. C2-ester was polymerized in a solution containing 10% (*v*/*v*) DMSO and 0.05% (*v*/*v*) TFA at 28 °C for 3 days at a peptide concentration of 11.1 mg/mL after annealing. The polymer was in the sol state after polymerization. Although the polymer was subjected to the addition of 10× phosphate-buffered saline (PBS) at pH 7.4 and incubated at 37 °C, the polymer, whose final concentration was 10.0 mg/mL, did not form a gel even after 1 h (Figure 3A). Conversely, individually prepared C2-amide at the same concentration formed a gel by polymerization.

To confirm *O–N* acyl migration in the polymer, the C2-ester polymer was subjected to the addition of 10× PBS, incubated at 37 °C for 1 h, treated with tris(2-carboxyethyl)phosphine (TCEP) to reduce disulfide bonds, and analyzed by HPLC (Figure 3B). The peak of C2-ester eluted at around 21 min decreased, and the peak of the corresponding amide form compound eluted at around 22 min increased by PBS treatment. The minor peaks at around 12 and 14 min were N- and C-terminus fragments, which were hydrolyzed products of C2-ester, respectively. However, there was little change in the amounts before and after the reaction, suggesting that they were not involved in *O–N* acyl migration. The ratio of *O–N* acyl migration was calculated by the peak areas in HPLC (Figure 3C). It was found that 80% and over 90% of C2-ester was converted to the amide form in 10 and 60 min, respectively.

It was revealed that C2-ester could not form a gel by itself even after the ester was converted to the amide. Because the C2-amide polymer formed a gel at the same concentration, prior polymerization of C2-ester is suggested to prevent sufficient triple helix formation from forming a gel.

### 2.4. Investigation of the Gel-Forming Conditions of the Copolymers of C2-Ester and Triple-Helical Peptides

**Figure 4:**
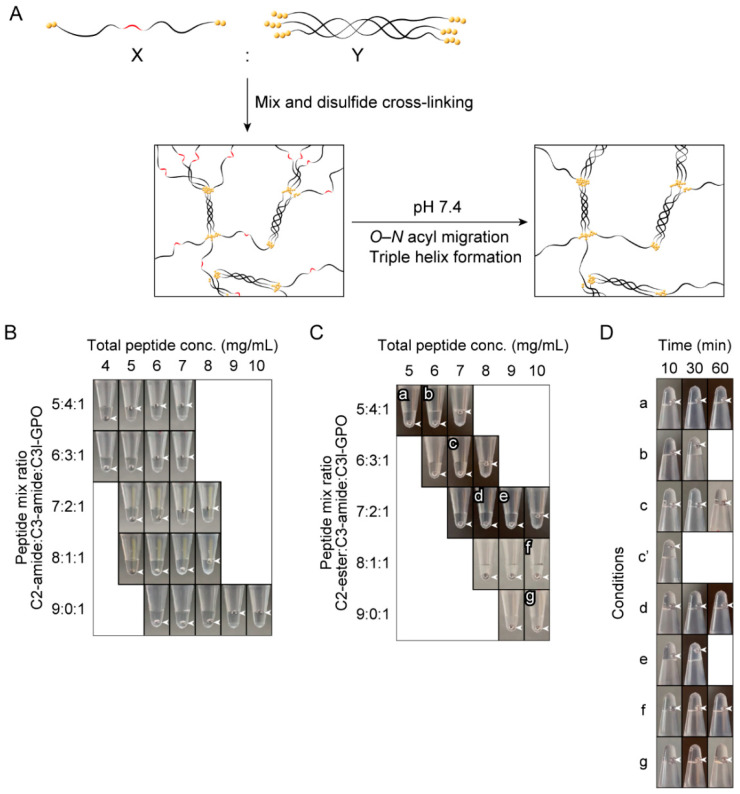
Investigation of the gel-forming conditions of the ester-amide copolymers. (**A**) Graphical image of the ester-amide copolymer in which the triple-helical content increases after *O–N* acyl migration at physiological pH. The ester form and amide form peptides were mixed in a certain ratio (X:Y) and polymerized. (**B**) Evaluation of gel formation using copolymers solely composed of the amide-form peptides. C2-amide, C3-amide, and C3l-GPO were copolymerized in 10% (*v*/*v*) DMSO for 4 days, and gel formation was evaluated by whether a stainless-steel ball was held on the interface of the polymer. (**C**) Evaluation of gel formation of copolymers composed of the ester and amide form peptides. C2-ester, C3-amide, and C3l-GPO were copolymerized, and gel formation was evaluated by the same procedure as above. (**D**) Evaluation of gel formation after *O–N* acyl migration. The copolymers obtained from (**C**), in which the adopted conditions are labeled **a**–**g**, were subjected to the addition of 10× PBS and incubated at 37 °C. Gel formation was evaluated by whether the stainless-steel ball was held on top of the tube after turning the tube upside down. The polymer of **c’** was 7.5 mg/mL polymer with the same peptide composition as c. The arrowheads indicate the positions of the stainless-steel balls.

It is considered that the polymer composed solely of C2-ester cannot form sufficient triple helices for gel formation even after *O–N* acyl migration. Therefore, we attempted to increase the triple-helical content in the polymer by copolymerizing other triple-helical peptides with C2-ester (Figure 4A). For this purpose, 2 peptides composed of 12 or 14 repeats of Pro-Hyp-Gly, 2 Cys residues at each terminus, and an Arg residue in the middle of the sequence were prepared (C3-amide and C3l-GPO, Table 1, Figures S1–S3). The Arg residue was introduced to prevent possible lateral aggregation of the Pro-Hyp-Gly repeating peptide [30].

First, to determine the conditions under which the polymer forms a gel after *O–N* acyl migration, the peptide composition, and concentration were investigated using the amide form peptides (C2-amide, C3-amide, and C3l-GPO). The amount of C3l-GPO was fixed at 10% of the total peptide. Copolymers with different ratios of C2-amide and C3-amide were prepared, and the concentration at which the polymer forms a gel was investigated (Figure 4B). It was found that the minimum gel-forming concentrations of the 5:4:1, 6:3:1, 7:2:1, 8:1:1, and 9:0:1 C2-amide:C3-amide:C3l-GPO copolymers were 5, 6, 7, 8, and 9 mg/mL, respectively.

Next, to investigate the compositions and concentrations at which the polymers were in the sol state before *O–N* acyl migration, copolymers of C2-ester, C3-amide, and C3l-GPO were prepared, and their gel formation was evaluated (Figure 4C). The 5:4:1, 6:3:1, and 7:2:1 C2-ester:C3-amide:C3l-GPO copolymers were in the sol state at concentrations of less than 7, 8, and 10 mg/mL, respectively, while the 8:1:1 and 9:0:1 C2-ester:C3-amide:C3l-GPO copolymers were in the sol state even at 10 mg/mL.

The copolymers in the sol state at relatively high concentrations (labeled **a**–**g** in Figure 4C) were examined for *O–N* acyl migration-induced gel formation at pH 7.4. The results of gel formation evaluation after the addition of 10× PBS to each polymer are shown in Figure 4D. The sols transformed to gels within 30 min for the 5:4:1 (**b**) and 7:2:1 (**e**) C2-ester:C3-amide:C3l-GPO copolymers at 6 and 9 mg/mL, respectively.

To determine the conditions under which the copolymers rapidly form gels after the pH shift, we also investigated the concentration of the 6:3:1 C2-ester:C3-amide:C3l-GPO copolymer. At a concentration of 7.5 mg/mL, the copolymer was in the sol state after polymerization and formed a gel within 10 min after the addition of 10× PBS (labeled **cʹ** in Figure 4D). In addition, the polymer formed a gel by injection into PBS (Movie S1). This revealed a collagen-like peptide polymer that formed a gel within 10 min through a sol–gel transition triggered by *O–N* acyl migration at pH 7.4. The surface structure of the resulting gel was observed by scanning electron microscopy (SEM) (Figure S4). In contrast to the native type I collagen gel, no fibrillar structures or pore structures were observed in the polymer gel. The structure was very similar to the gel developed in our previous study [23].

### 2.5. Application as a 3D Scaffold for Cell Culture

We investigated whether the collagen-like peptide polymer optimized above could be applied to 3D cell culture and whether it could regulate specific behaviors of cells by the action of collagen-derived bioactive sequences incorporated into the polymer. We used the Madin–Darby canine kidney (MDCK) cell line, a canine renal tubular epithelial cell line, as a typical model for the formation of 3D structures in 3D matrices. MDCK cells are known to form cysts when cultured in type I collagen gels [31,32]. We investigated whether MDCK cells also formed cysts in the collagen-like peptide polymer gels containing certain collagen-derived bioactive sequences.

Peptides containing each of the three bioactive sequences were prepared (Table 1, Figures S1–S3). The Nle residue introduced into C3l-GVXGFO was used as an oxidation-resistant Met analog. This sequence has been reported to function as a ligand for DDR [33]. The copolymers composed of C2-ester, C3-amide, and either one of C3l-GPO, C3l-GFOGER, C3l-GVXGFO, or C3l-KGHRGF (called the GPO polymer, GFOGER polymer, GVXGFO polymer, and KGHRGF polymer, respectively) at a ratio of 6:3:1 and a concentration of 7.5 mg/mL were prepared by the same procedure as above. These copolymers were all in the sol state after polymerization.

To embed MDCK cells in the polymer gel, the polymer solution after oxidation was first neutralized to weakly basic pH by adding 10× Dulbecco’s modified Eagle’s medium (D-MEM), and the dispersed MDCK cells after trypsinization were mixed at a volume ratio of 17:2:1. The final concentration of the polymer was 6.4 mg/mL. The mixture was then immediately transferred to molds placed in cell-culture dishes and incubated at 37 °C for gel formation. As a control, type I collagen acidic solution was mixed with 10× D-MEM and MDCK cells, and it formed a gel on a cell-culture dish according to the same procedure. The final collagen concentration of the gel was 2.3 mg/mL. After 30 min, D-MEM containing 10% (*v*/*v*) fetal bovine serum (FBS) was added onto the gel, and the cells were cultured at 37 °C. After 1 week, the cells were fixed, and the nuclei and F-actin were observed by fluorescent staining. Most of the cells in each gel formed a spherical structure with a diameter of less than 100 µm. Small numbers of larger cell aggregates were also found. Typical morphologies of larger aggregates and all the smaller aggregates are shown in Figures S5 and S6. The numbers of the smaller aggregates were 39, 51, 47, 40, and 32 for type I collagen gel, GPO polymer gel, GFOGER polymer gel, GVXGFO polymer gel, and KGHRGF polymer gel, respectively. The numbers in the polymer gels were comparable to that in type I collagen gel, indicating that the peptide polymer gel had little cytotoxicity. It was confirmed that the collagen-like peptide polymer gels are compatible with the 3D cell-culture system.

**Figure 5:**
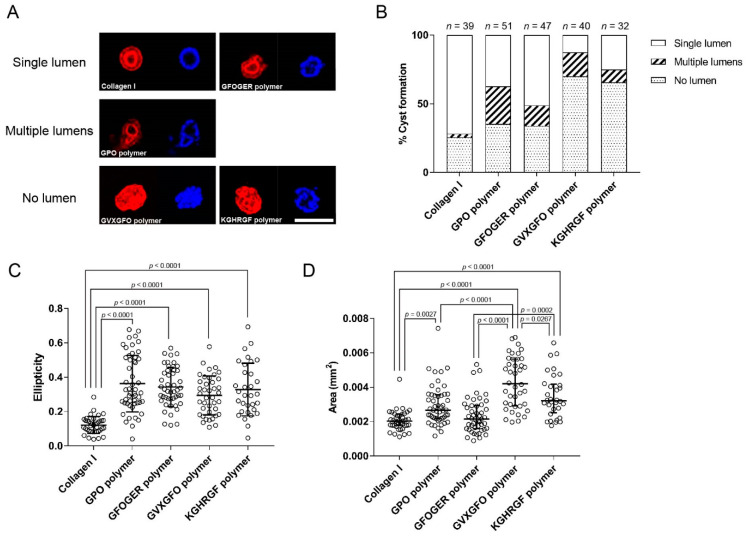
Morphological analysis of the MDCK-cell spheres in the gels. (**A**) Typical morphologies of the MDCK-cell spheres in the gels. The cross-sectional images were obtained by confocal fluorescence microscopy. F-actin and the nuclei were stained in red and blue, respectively. The scale bars indicate 100 µm. (**B**) Quantitative analysis of cyst formation in the gels. The cell spheres with a diameter of less than 100 µm were classified as single-luminal, multi-luminal, or non-luminal spheres. The ellipticity (**C**) and cross-sectional area (**D**) of the spheres were measured. The significance of the differences was assessed by non-parametric one-way analysis of variance and the post-hoc Tukey test, and all the *p* values < 0.05 are shown in the figures.

The cell spheres smaller than 100 µm in diameter were morphologically classified into three categories: cysts with a single lumen, cysts with more than one lumen, and aggregates without a lumen. Typical cross-sectional images of the cell aggregates in this experiment are shown in Figure 5A. The luminal spheres showed the localization of F-actin along the lumen, indicating the formation of apical surfaces inside the sphere. The presence or absence of lumens and the number of lumens of the type I collagen gel and collagen-like peptide polymer gels were compared (Figure 5B). In terms of lumen formation, the type I collagen, GPO polymer, and GFOGER polymer gels were comparable, and fewer spheres formed lumens in the GVXGFO polymer and KGHRGF polymer gels. From comparison of the number of lumens, most of the luminal spheres were single-luminal cysts in the type I collagen gel. Conversely, the ratios of single-luminal cysts and multi-luminal cysts were similar in the GPO polymer gel without any specific bioactive sequences. In the GFOGER polymer gel, the ratio of the single-luminal cyst was higher than that in the GPO polymer gel, and the cyst-forming activity was the highest among the collagen-like peptide polymer gels.

The ellipticity and area of the cross-sections of the spheres were measured (Figure 5C,D). The cross-sections of the spheres in the type I collagen gel were close to perfect circles, whereas those of the spheres formed in all the collagen-like peptide polymer gels were distorted, and their ellipticity was significantly higher (Figure S5). The ellipticity seemed to depend not on the bioactive sequences incorporated into the polymers but on the mechanical properties of the scaffolds. The relationship between the rheological properties of the gels and cell behavior is desirable to be investigated in the future.

Overall, the cross-sectional area of the sphere was inversely correlated with the single-luminal cyst-forming activity. The cross-sectional areas of the spheres in the type I collagen gel were significantly smaller than those in the collagen-like peptide polymer gels, except for the GFOGER polymer gel. Comparing the introduced bioactive sequences, the cross-sectional areas of the spheres in the GVXGFO polymer gel were significantly larger than those in the other collagen-like peptide polymer gels, and those of the spheres in the KGHRGF polymer gel were significantly larger than those in the GFOGER polymer gel.

Overall, these results indicated that the integrin α2β1-binding sequence promotes cyst formation, including polarization of MDCK cells. The contribution of integrin α2β1 to cyst formation is supported by previous studies that showed that cyst formation of MDCK cells in type I collagen gels is inhibited by integrin α2β1 knockdown and specific inhibitory antibodies [34,35]. In addition, previous studies using a 3D culture system of synthetic matrices conjugated with the Arg-Gly-Asp peptide, a ligand for integrin α_V_β3, have also shown that activation of integrin promotes MDCK-cell cyst formation [36,37].

In contrast to the GFOGER sequence, cyst formation in MDCK cells is suppressed by the Arg-Gly-Gln-Hyp-Gly-Val-Nle-Gly-Phe-Hyp (RGQOGVXGFO) sequence incorporated into C3l-GVXGFO and Lys-Gly-His-Arg-Gly-Phe-Ser-Gly-Leu (KGHRGFSGL) sequences incorporated into C3l-KGHRGF. These sequences bind to cell surface receptors and soluble proteins: DDR [7], VWF [8], and SPARC [9,10] for RGQOGVXGFO, and heparan sulfate of syndecans [11] and PEDF [12,13] for KGHRGFSGL. The mechanisms by which these collagen-binding proteins regulate the polarity and cyst formation need to be further investigated in the future.

In this study, three bioactive sequences derived from natural collagen were individually incorporated into artificial collagen-like polymers, but it is also possible to incorporate several bioactive sequences in combinations. The cooperative or competitive effects of multiple collagen receptors could be investigated by taking advantage of the 3D cell-culture system.

## 3. Materials and Methods

### 3.1. Cell Culture

The MDCK cells were purchased from the American Type Culture Collection (ATCC, Manassas, VA, USA). The cells were cultured in D-MEM (Fujifilm Wako Pure Chemical Industries, Osaka, Japan) containing 10% (*v*/*v*) FBS (Thermo Fisher Scientific, Waltham, MA, USA), 100 units/mL penicillin, and 100 μg/mL streptomycin (Sigma-Aldrich, St. Louis, MO, USA). The cells were maintained at 37 °C in a humidified 5% CO_2_/air atmosphere.

### 3.2. Peptide Synthesis

The peptides were manually synthesized according to the standard Fmoc-based solid-phase method on 2-chlorotrityl chloride resin (Peptide Institute, Osaka, Japan). The coupling reaction was performed at room temperature for 2 h with 3–5 equivalents of Fmoc-amino acid, DIC, and HOBt in *N*,*N*-dimethylformamide. The Fmoc group was deprotected using 20% (*v*/*v*) or 30% (*v*/*v*) piperidine/DMF for 15 min. Fmoc-Pro-Hyp-Gly-OH (Watanabe Chemical Industries, Hiroshima, Japan) and Boc-Ser(Fmoc-Gly)-OH (Iris Biotech, Bayern, Germany) were used for incorporation of Pro-Hyp-Gly repeats and the glycylserine *O*-acyl isopeptide unit, respectively. The Fmoc group of the amino acid residue next to the glycylserine *O*-acyl isopeptide unit was deprotected using 25% (*v*/*v*) 1-methylpyrrolidine, 2% (*v*/*v*) hexamethyleneimine and 3% (*w*/*v*) HOBt in *N*-methylpyrrolidone:DMSO (1:1) [38]. The peptides were deprotected and cleaved from the resin by TFA/*m*-cresol/thioanisole/ethanedithiol/H_2_O (80:5:5:5:5, *v*/*v*) at room temperature for 4 h. The crude peptides were purified by RP-HPLC on a Cosmosil 5C_18_-AR II column (Nacalai Tesque, Kyoto, Japan) and identified by matrix-assisted laser desorption–ionization time-of-flight mass spectrometry (Autoflex III, Bruker, Billerica, MA, USA) or electrospray ionization mass spectrometry (AB Sciex, Framingham, MA, USA).

### 3.3. CD Analysis

The CD spectra were recorded with a J-820 CD spectropolarimeter (JASCO, Tokyo, Japan) equipped with a Peltier thermal controller. The intensity of the spectra was converted into the mean residue weight ellipticity ([θ]_MRW_). The peptides were dissolved in degassed 0.05% (*v*/*v*) TFA(aq) containing 10 mM TCEP, followed by annealed at 4 °C overnight after heating at 95 °C for 5 min. The thermal denaturation curve of the triple helix was obtained by monitoring [θ]_MRW_ at 225 nm ([θ]_MRW,225_) with increasing temperature from 4 to 95 °C at a rate of 18 °C h^−1^.

### 3.4. Preparation of the Peptide Polymers and Evaluation of Gel Formation

The peptides were dissolved in degassed 0.05% (*v*/*v*) TFA(aq) and annealed, as described above. DMSO was added to the peptide solution to a final concentration of 10% (*v*/*v*), and the solution was stood at 28 °C for 4 days for polymerization. To induce *O–N* acyl migration of *O*-acyl isopeptide in the polymer, 10× PBS (50 mM phosphate buffer (pH 7.4), 100 mM NaCl) was added to the polymer solution, followed by incubation at 37 °C for 5–60 min and dilution with 0.05% (*v*/*v*) TFA(aq). TCEP was then added to the solution at the final concentration of 50 mM, and the solution was heated at 95 °C for 3 min, followed by RP-HPLC analysis.

Gel formation was evaluated by gently placing a stainless-steel ball (SUS440C, diameter = 1.5 mm; Funabe Seiko, Hyogo, Japan) on the interfaces (Figure 4B,C). *O–N* acyl migration-induced gel formation was similarly assessed by the position of the ball after turning the tube with the ball at the bottom upside down after incubation at 37 °C (Figure 4D).

For a supplemental movie (Movie S1), a 7.5 mg/mL collagen-like peptide polymer solution of C2-ester:C3-amide:C3l-GPO (6:3:1) was prepared using 0.05% (*v*/*v*) TFA(aq) containing 10% (*v*/*v*) glycerol and 10% (*v*/*v*) DMSO as a solvent. The polymer solution was injected by syringe (22G) into 0.01% (*w*/*v*) trypan blue stained 0.05% (*v*/*v*) TFA(aq) or PBS in a glass vial and kept at room temperature for 30 min.

### 3.5. SEM

C2-ester:C3-amide (1:1, total conc. = 7 mg/mL) copolymer was prepared in the same procedure as above. The polymer was mixed with 10× D-MEM (prepared by dissolving powder mix and NaHCO_3_, Sigma-Aldrich), and 20 µL of the mixture was added onto a coverslip and incubated at room temperature for 30 min for gel formation. Type I collagen gel (2.5 mg/mL) was prepared by mixing collagen solution with 10× PBS and incubation at 37 °C for 30 min. Gels were dehydrated in graded alcohol series of H_2_O/methanol (50–100%) and methanol/*tert*-butanol (50–100%), and the following lyophilization. All samples were coated with platinum and observed by S-3000N (Hitachi, Tokyo, Japan).

### 3.6. Three-Dimensional Cell Culture

The MDCK cells were detached with 0.05% (*w*/*v*) trypsin/0.53 mM ethylenediaminetetraacetic acid (Fujifilm Wako Pure Chemical Industries) and harvested by centrifugation (120× *g*, 4 min). The peptide polymer solution (7.5 mg/mL), 10× D-MEM, and the MDCK-cell suspension were mixed at a ratio of 17:2:1, and 30 µL of the mixture was added to a circular silicone mold (diameter = 4 mm, height = 1 mm) set on a glass-based 35 mm dish (Iwaki, Tokyo, Japan) and incubated at 37 °C for gel formation, followed by addition of 2 mL of D-MEM containing 10% (*v*/*v*) FBS. To prepare the type I collagen gel, 3 mg/mL type I collagen acidic solution (I-AC, Koken, Tokyo, Japan), DMSO, 10× D-MEM, and MDCK-cell suspension were mixed at a ratio of 15:2:2:1 and incubated at 37 °C. After culturing at 37 °C for 7 days, the cells were fixed with 4% *p*-formaldehyde/phosphate buffer (Fujifilm Wako Pure Chemical Industries) overnight, permeabilized with 0.1% (*v*/*v*) Triton X-100/PBS for 15 min, and blocked with 1% (*w*/*v*) skim milk/PBS for 1 h. The nuclei and F-actin were stained by Hoechst 33342 and phalloidin-iFluor conjugate (Cayman Chemical, Ann Arbor, MI, USA) for 3 h. The cells were observed by confocal fluorescence microscopy (FV-1000, Olympus, Tokyo, Japan) after washing with PBS.

For quantitative analysis of cyst formation, the spheres were classified as single-luminal, multi-luminal, or no-luminal spheres using cross-sectional images. The area and ellipticity of the cell spheres were measured by ImageJ software (version 1.52u) [39]. The cross-sectional area was defined as the largest area among the photographs taken of each sphere every 3 µm in the horizontal direction. The ellipticity was calculated by ellipticity = 1 − (shortest diameter/longest diameter) for the photograph with the largest cross-sectional area. All the analyses were performed blindly by a third person.

## 4. Conclusions

We have developed a collagen-like peptide polymer that forms a gel within 10 min by triple-helix formation triggered by *O–N* acyl migration under basic conditions by incorporating a glycylserine *O*-acyl isopeptide unit into the collagen-like peptide sequence. This allowed encapsulation and culturing of cells in the 3D gel, which were not possible using our previously developed similar artificial collagen gels [22]. Furthermore, we demonstrated that cyst formation with the polarization of epithelial cells can be controlled by incorporating the bioactive sequences of collagen into the artificial collagen gel.

This polymer-based 3D cell-culture system is expected to allow investigation of the mechanism by which collagen-binding molecules control the behavior of cells, including proliferation, differentiation, and polarization. In the biomedical field, applications of injectable gels, such as scaffolds for in vitro stem cell culture and carriers for cell transplantation, are also expected.

## Figures and Tables

**Table 1:** List of the peptides used in this study.

Peptide	Sequence
C2-ester	H-Cys-Cys-(Host)-Pro-Hyp-Z-Hyp-Gly-(Host)-Cys-Cys-OH
C2-amide	H-Cys-Cys-(Host)-Pro-Hyp-Gly-Ser-Hyp-Gly-(Host)-Cys-Cys-OH
C3-amide	H-Cys-Cys-Cys-(Host)-Pro-Hyp-Gly-Pro-Arg-Gly-(Host)-Cys-Cys-Cys-OH
C3l-GPO	H-Cys-Cys-Cys-(Host)-Pro-Pro-Gly-Pro-Pro-Gly-Pro-Arg-Gly-Pro-Pro-Gly-(Host)-Cys-Cys-Cys-OH
C3l-GFOGER	H-Cys-Cys-Cys-(Host)-Pro-Pro-Gly-Phe-Hyp-Gly-Glu-Arg-Gly-Pro-Pro-Gly-(Host)-Cys-Cys-Cys-OH
C3l-GVXGFO	H-Cys-Cys-Cys-(Host)-Pro-Arg-Gly-Gln-Hyp-Gly-Val-Nle-Gly-Phe-Hyp-Gly-(Host)-Cys-Cys-Cys-OH
C3l-KGHRGF	H-Cys-Cys-Cys-(Host)-Pro-Lys-Gly-His-Arg-Gly-Phe-Ser-Gly-Leu-Hyp-Gly-(Host)-Cys-Cys-Cys-OH

Z is the glycylserine *O*-acyl isopeptide unit. Host is (Pro-Hyp-Gly)_5_. Nle (X) is a norleucine residue.

## Data Availability

The data presented in this study are available in the article or Supplementary Material.

## Supplementary Materials

The following are available online at https://www.mdpi.com/article/10.3390/ijms23031584/s1.
