# Supplementary material for: Synthetic Collagen-like Polymer That Undergoes a Sol–Gel Transition Triggered by O–N Acyl Migration at Physiological pH
Source: Int J Mol Sci. 2022 Jan 29;23(3):1584. doi: 10.3390/ijms23031584 (PMC8835898; doi:10.3390/ijms23031584)
Supplement: Supplementary file 1 [file ijms-23-01584-s001.zip › ijms-1534755-supplementary.pdf]

Supplemental materials

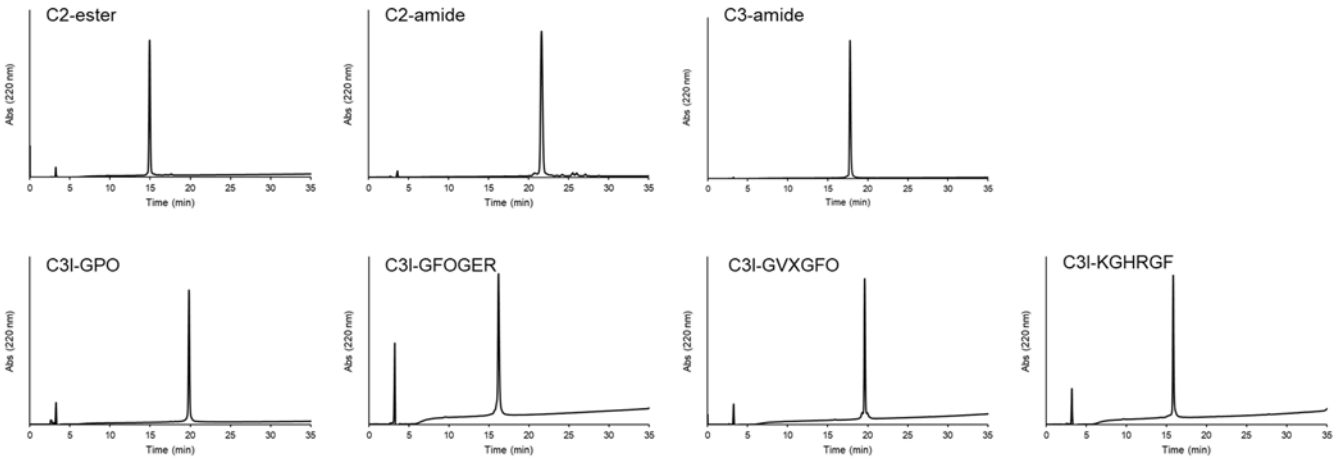

Figure S1. RP-HPLC analysis of the peptides. The peptides were analyzed on a Cosmosil $5C_{18}$ AR-II column with the linear gradient of acetonitrile/water (10%–20% for **C2-amide**, 10%–30% for **C2-ester**, **C3-amide**, and **C3l-GPO**, and 10%–40% for **C3l-GFOGER**, **C3l-GVXGFO** and **C3l-KGHRGF** in 30 min) at 60 °C at a flow rate of 1 mL min^{-1}.

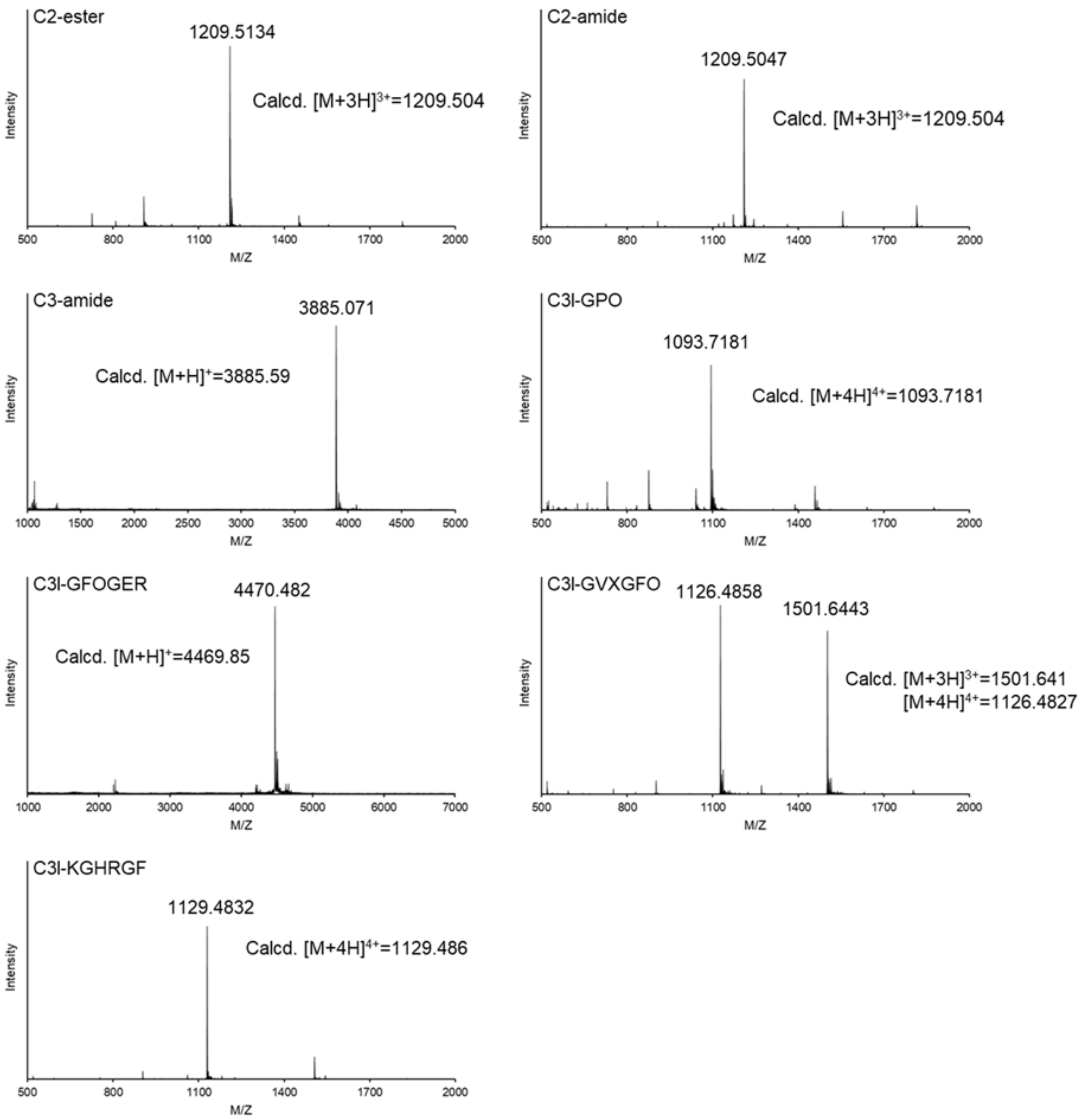

Figure S2. Mass analysis of the peptides. **C3m-amide** and **C3l-GFOGER** were analyzed by matrix-assisted laser desorption–ionization time-of-flight mass spectrometry (MS) using α-cyano-4-hydroxycinnamic acid as a matrix, and the other samples were analyzed by electrospray ionization MS.

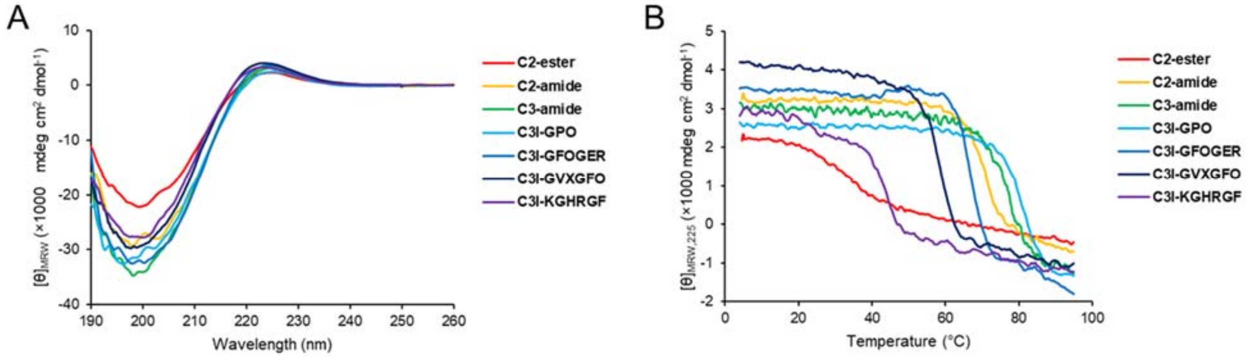

Figure S3. CD analysis of the peptides. (A) CD spectra of the peptides at 4 °C. The peptides were annealed in 0.05% TFA(aq), and the spectra were measured in the presence of 10 mM TCEP. (B) Monitoring of the CD signal at 225 nm with increasing temperature from 4 to 95 °C. Data of **C2-ester** and **C2-amide** are the same in Figure 2.

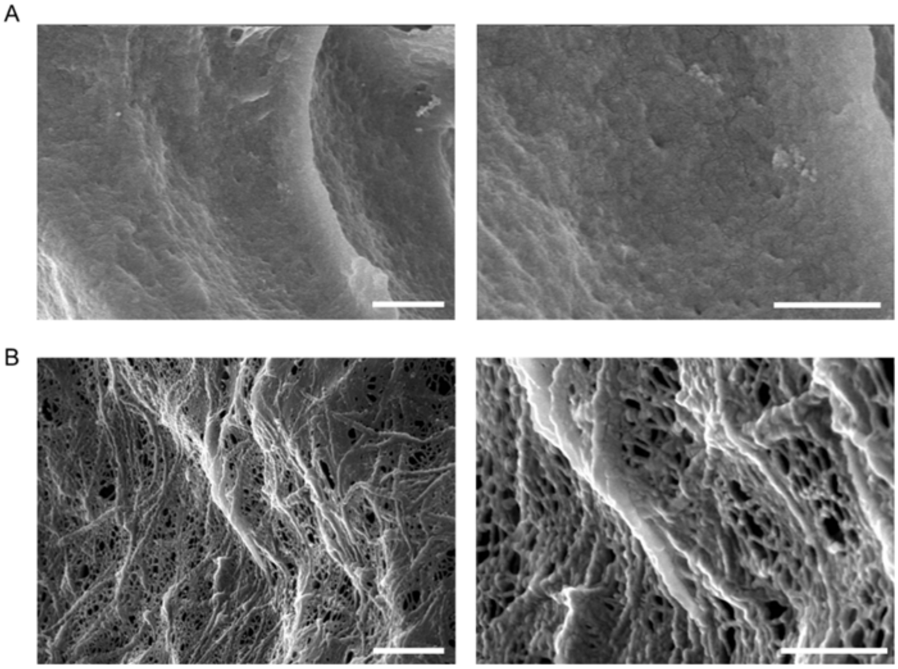

Figure S4. SEM images of the peptide polymer gel and type I collagen gel. Platinum-coated surface of **C2-ester** and **C3-amide** copolymer after sol–gel transition (A) and type I collagen gel (B) was observed at two different magnifications [× 20,000 (left) and × 60,000 (right)]. Scale bars in left and right panels indicate 1 μm and 500 nm, respectively. **C2-ester**:**C3-amide** (1:1) copolymer (total concentration = 7 mg/mL) was mixed with 10× D-MEM, and incubated at room temperature for 30 min for gel formation. Type I collagen gel (2.5 mg/mL) was prepared by mixing 3 mg/mL acid soluble type I collagen and 10× PBS followed by incubation at 37°C for 30 min.

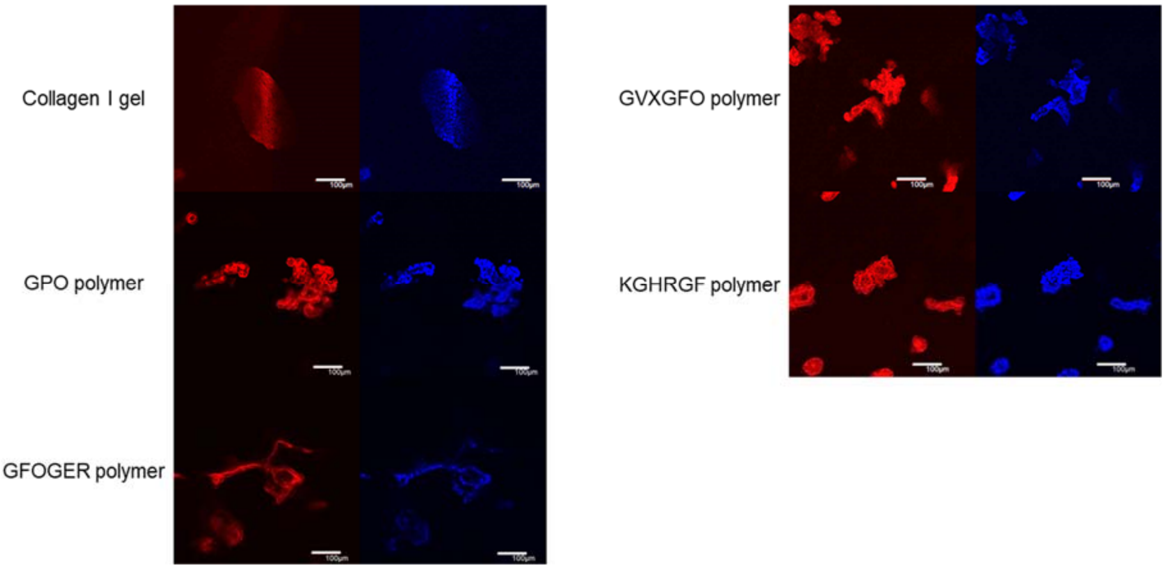

Figure S5. Fluorescence images of MDCK cell aggregations. MDCK cell aggregations with size of larger than 100 μm were observed by confocal fluorescence microscopy. The nuclei and F-actin were stained in blue and red, respectively. The scale bars indicate 100 μm.

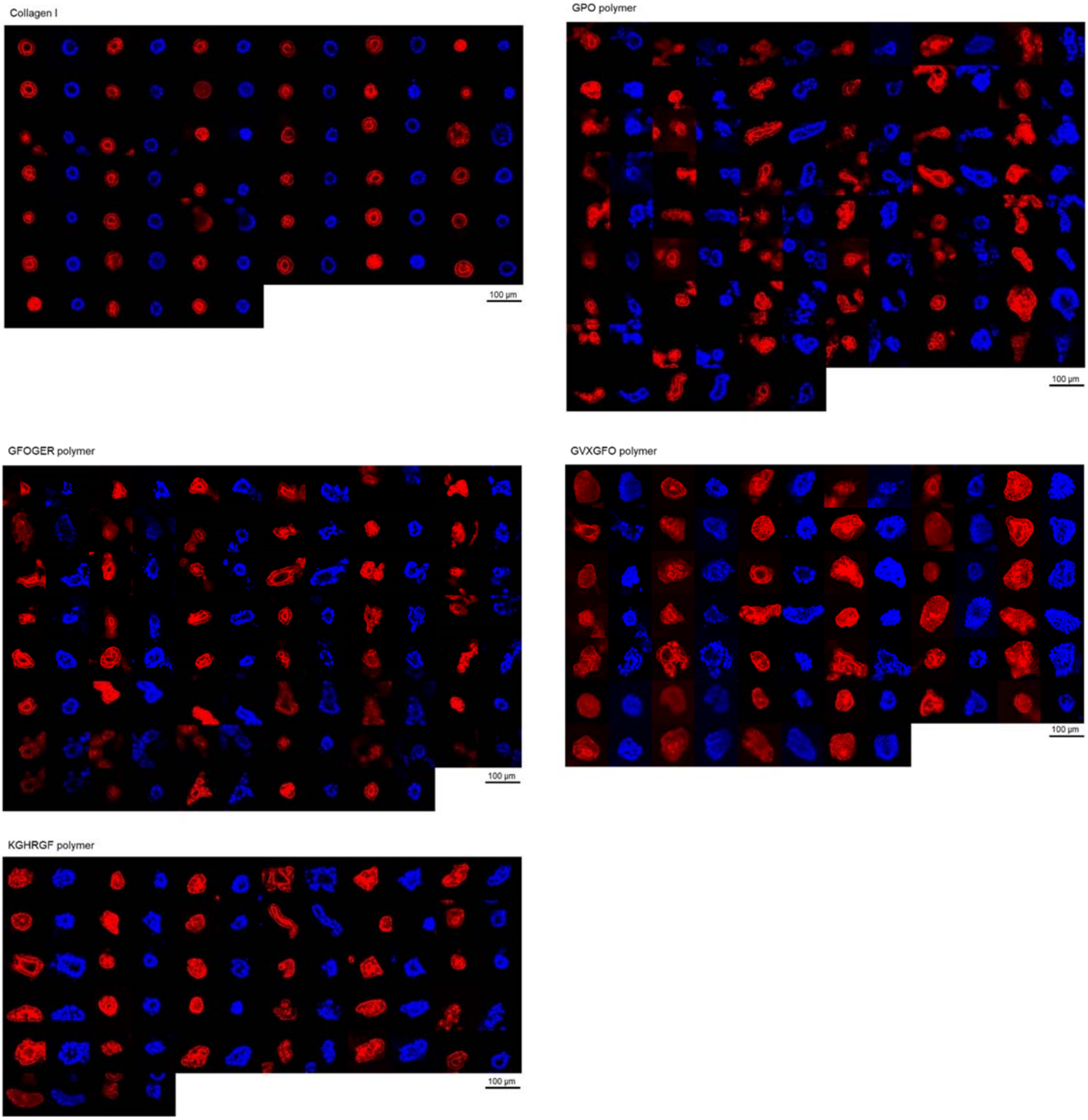

Figure S6. Fluorescence images of the MDCK-cell spheres. MDCK-cell spheres with diameter of less than 100 µm were observed by confocal fluorescence microscopy.
